# Reliability and validity of the Malay version of the drive-thru community pharmacy service questionnaire and the Malaysian public’s awareness, attitudes, and perceptions of drive-thru community pharmacy during COVID-19

**DOI:** 10.1186/s40545-023-00666-6

**Published:** 2023-11-28

**Authors:** Bayan F. Ababneh, Siew Chin Ong, Louai Alsaloumi, Hisham Z. Aljamal, Rabia Hussain

**Affiliations:** 1 Discipline of Social and Administrative Pharmacy, School of Pharmaceutical Sciences, Universiti Sains Malaysia, Penang, Malaysia; 2 Discipline of Clinical Pharmacy, Faculty of Pharmacy, Near East University, Northern Cyprus, Turkey; 3 Discipline of Orthopedics, Dr. Sulaiman AL Habib Medical Group, Al Khobar, Saudi Arabia

**Keywords:** Reliability and validity, Awareness, Public, Drive-thru community pharmacy services, Perceptions, Pharmacy, COVID-19

## Abstract

**Introduction:**

Understanding general public's experiences of using drive-thru pharmacies during COVID-19 in different countries is imperative for establishing these services by standardizing global guidelines for drive-thru pharmacies. The objectives of this study were to validate a Malay version of the drive-thru community pharmacy service questionnaire for use among Malaysians and to obtain a better understanding of the public’s awareness, attitudes, and perceptions of drive-thru community pharmacy service during COVID-19.

**Methods:**

This study was a cross-sectional study, conducted online using a Google form survey using a convenient sampling method among the Malaysian public. The English version of the drive-thru community pharmacy service questionnaire was translated into the Malay language according to international guidelines. The content and face validity of the questionnaire were examined by experts. Then, the questionnaire was pilot tested on 15 native speakers. Reliability was assessed using Cronbach’s alpha coefficients. The test–retest reliability was measured with Cohen’s κ coefficient.

**Results:**

A total of 519 participants completed the questionnaire. Face and content validity were satisfactory, as noticed by both the experts and pilot study participants. For test–retest reliability (32 participants), most perception statements had very good coefficient agreement values. Cronbach’s alpha of the perception part was 0.833, indicating strong internal consistency. The median age of study participants was 50.0 (IQR = 31.0) and about half of them were females (50.3%, *n* = 261). Despite 20.4% (*n* = 106) of the participants reported that the drive-thru community pharmacy service was available in their cities, only 10.4% (*n* = 54) reported using it. Most participants were in favor of introducing drive-thru services in community pharmacies throughout the country. Drive-thru community pharmacies, according to more than half of the participants (*n* = 394), would be beneficial to the public. Participants acknowledged that community pharmacies with drive-thru services were useful during the COVID-19 and quarantine periods due to the enhanced social distance 43.5% (*n* = 226), reduced the spread of the COVID-19 virus 47.0% (*n* = 244), and relieved pressure on other healthcare settings 38.2% (*n* = 198).

**Conclusions:**

The translated questionnaire was valid and reliable in assessing the perceptions toward drive-thru community pharmacy service during COVID-19 in Malaysia. The participants expressed good awareness and favorable attitudes and perceptions toward drive-thru community pharmacy service during COVID-19. Furthermore, they perceived those services helped to increase social isolation and stop the COVID-19 virus from spreading.

**Supplementary Information:**

The online version contains supplementary material available at 10.1186/s40545-023-00666-6.

## Introduction

One of the safety measures that was issued by the World Health Organization (WHO) during COVID-19, involved establishing a social distance of at least one meter between individuals [1]. Mandatory lockdowns and fear of being infected changed physical ways of obtaining everyday necessities favor safer ways [2]. These ways generally require little to no personal interaction, such as online shopping, or drive-thru services [3, 4]. The drive-thru approach was an appropriate option to fulfill the demands of consumers with a social distancing tactic [3–5].

Many countries, including the United States of America [6], the United Kingdom [7], Croatia [8], Malaysia [9, 10], Jordan [11], Taiwan [12], Qatar [13], Australia [14], and others [15, 16] have implemented the drive-thru pharmacy service. In addition, it was used to shorten wait times in pharmacies, enhance the accessibility and provision of healthcare services for the specific population, and most recently, to increase safety during COVID-19 [16, 17].

In Malaysia, drive-thru pharmacy service was part of the Pharmacy Value Added Service (VAS) provided by the Malaysian Pharmaceutical Services Division, Ministry of Health Malaysia in 2003 [9]. As a pilot project, it was first launched in 2008 in Penang General Hospital [10]. The drive-thru pharmacy service was then implemented in 18 hospitals and 18 health clinics in 2014 as an effort to solve parking problems and relieve congestion in the pharmacy waiting area, according to the Pharmaceutical Services Division of the Ministry of Health of Malaysia [18].

Two studies were conducted in Malaysian hospitals to assess the drive-thru pharmacy service [19, 20]. First study was conducted in the Queen Elizabeth Hospital (QEH), Kota Kinabalu, and indicated that patients who utilized the drive-thru pharmacy service at QEH were satisfied with the service [19]. Second study was from the Hospital Raja Perempuan Zainab II (HPRZ II), where patients were aware of the availability of a drive-thru pharmacy service and emphasized the value of utilizing this service [20].

Customers at community pharmacies in Jordan have reported positive experiences with drive-thru services. They verified that such services are quick and convenient [21]. In addition, the first drive-thru pharmacy in Taiwan proved to provide quick and efficient service in comparison with typical pharmacy services [12]. Drive-thru pharmacies, however, may limit interactions between the pharmacist and the patient, which has a significant impact on the counseling process. The evaluation of awareness, perception, and barriers among Jordanian pharmacists provides additional support for this evidence [11]. On the other side, drive-thru pharmacies can offer convenient medication dispensing and address the issue of unavailable parking spaces, which will improve customer satisfaction [11].

The COVID-19 pandemic increased the need for medication sales and production, and consumers strongly preferred quick-serving pharmaceutical services, such as drive-thru service [15]. Learned experiences from utilizing drive-thru pharmacies during COVID-19 in many countries could help in promoting the benefits of introducing these services by creating standardized guidelines for drive-thru pharmacies [16]. A survey-based study was conducted in Saudi Arabia to determine the demand for drive-thru pharmacy services during COVID-19; the findings indicated a high need to support the community pharmacy with drive-thru pharmacy services [22]. In response to a need during the COVID-19 pandemic, Superbig Kubang Kerian Pharmacy opened the first community pharmacy with drive-thru services on February 5th, 2022. Customers were more likely to visit community pharmacies to purchase vitamins, hygiene items, masks, and even their regular medications to avoid going to hospitals and clinics and to increase social distance [23]. This community drive-thru pharmacy could decrease pressure on the hospital pharmacies [23].

Understanding the Malaysian public’s awareness, attitudes, and perceptions of drive-thru community pharmacies during COVID-19 is important to assess this service. The original English version of the tool was developed and validated by the research team [24, 25]. However, the valid tool was not available in Malay language. Furthermore, it could be challenging to effectively represent the perceptions, and attitudes of the target population due to differences in populations, languages, and cultures [26].

Moreover, the majority of Malaysians speak Malay as their mother tongue. The Malay language is widely spoken throughout Southeast Asia, including Malaysia, Indonesia, Singapore, and some regions of Thailand. Nevertheless, English is a second language in Malaysia, where 50% of the population can read and write it [27]. Previous studies that assessed general public’s attitudes, awareness, and perceptions of certain healthcare services or health conditions in Malaysia proved that translated tools were helpful [28–31].

Therefore, the availability of the instrument in the Malay language would ensure that the instrument can target the population that has been excluded by the English version and be used in a larger population for more generalizability. Therefore, this study aimed to validate a Malay version of the drive-thru community pharmacy service questionnaire for use among Malaysians and to obtain a better understanding of the Malaysian public’s awareness, attitudes, and perceptions of drive-thru community pharmacy service during COVID-19.

## Methods

### Study design and setting

A cross-sectional study was conducted using a convenient sampling method in Malaysia between May 2022 and June 2022. The data collecting tool was a self-administered survey using an online Google form. The survey was originally developed after a thorough literature review [11, 19–22], some suggestions by community pharmacists practicing in Malaysia, and factor analysis (exploratory factor analysis and confirmatory factor analysis) for the developed English version survey [24]. The survey was later distributed to the participants by research assistants (undergraduate pharmacy students at Universiti Sains Malaysia) via social media platforms, such as WhatsApp, Instagram, and Telegram applications. The estimated time taken to complete answering the survey was approximately 5–7 min. To assess the test–retest reliability of the questionnaire, the questionnaire was administered a week after the first set of the questionnaire.

### Participants

Participants were recruited throughout Malaysia. The inclusion criteria for participants were: Malaysian adults aged 18 years and above, Malaysian resident, read and understand Malay, and had access to the internet via computer or smartphone to answer the instrument through online platforms. The exclusion criteria were foreigners, aged below 18 years or had no access to the internet via computer or smartphone to answer the instrument through online platforms.

### Sample size

The sample size was calculated using the following formula [32], which revealed that the sample size needed to be at least 363 participants $n= z 2\times \hat{\rho} (1-\hat{\rho} ) \div \varepsilon 2$, $z$ is the *z* score, $\varepsilon$ is the margin of error, $n$ is the population size, and $\hat{\rho}$ is the population proportion. *Z* for a confidence level of 95% is 1.96. The margin of error is 5%. Assume a population proportion of 0.6, since a Malaysian study found that 60% of people were aware of drive-thru service [20] and unlimited population size. An additional 43% of the participants were recruited (519) to account for any missing data.

### Ethical considerations

Study approval was obtained from the Human Research Ethics Committee of Universiti Sains Malaysia (USM) (Reference code: USM/JEPeM/21110755). Before beginning the first section of the instrument, those who accepted to participate in the study, electronically signed the consent form.

### Survey instrument

The survey instrument was administered in the Malay version. The survey instrument was divided into three main sections with 49 items. Items for the first section and second section which were socio-demographic information and attitudes of the general public toward drive-thru community pharmacy service, respectively, were designed as open-ended questions and multiple-choice questions. Items for the third section discussed the perceptions, believed advantages and disadvantages toward drive-thru community pharmacy service, and were designed in Likert Scale-type questions (5 = strongly agree, 4 = agree, 3 = neutral, 2 = disagree, and 1 = strongly disagree), with a reverse coding scheme taken into account for negative statements and for the disadvantages (1 = strongly agree, 2 = agree, 3 = neutral, 4 = disagree, and 5 = strongly disagree), to indicate the degree of agreement or disagreement of participants after they went through the respective statements in this section.

### Translation of the instrument

Using international guidelines, the English version of drive-thru community pharmacy service questionnaire was translated into the Malay language [33]. The questionnaire was translated independently by a group of professionals from language school at USM with a qualification of (Ph.D. Bahagian Bahasa Malaysia), then other translators translated the Malay version back into English. To assess the similarity, a comparison between the translated back version and the original version was conducted. In addition, to assess the satisfactory and appropriate concept, the content and face validity of the questionnaire was screened [34]. Experienced academic pharmacists at USM, one community pharmacist, and two bilingual physicians who were fluent in both English and Malay languages were invited to review the content and face validity of the questionnaire before distributing it to the participants. The goal was to ensure that the Malay versions were equivalent to the original English version in terms of its content, wording, and cognitive level and that the questionnaire was linguistically appropriate to Malaysians. Any amendments were made based on the received feedback and suggestions to obtain the final version. For the questionnaire's pilot testing, fifteen Malaysians who were native speakers and proficient in the Malay language were selected. They were asked about any confusion or difficulty they had understanding the questionnaire's items as well as any suggestions for prospective question rephrasing [35]. No suggestions or comments were received from them. To ensure the reliability of the instrument, internal consistency which measured how closely related the items were for each scale, was calculated using Cronbach’s alpha coefficient for perceptions score, with values of 0.70 and above indicating good internal consistency [36]. Both the English and the Malay languages versions of the study instrument are available in Additional file 1 and Additional file 2, respectively.

### Statistical analysis

Statistical methods were used to analyze the data, including the calculation of descriptive statistics, such as the frequency and percentage of categorical variables. Continuous data such as age and perceptions score were tested for normality by Kolmogorov–Smirnov test. Since the data did not support parametric assumptions the median and the interquartile range were reported.

Reliability was measured over time (test–retest reliability) and across items (internal consistency) [37]. The reliability of the instrument was tested with Cohen’s Kappa statistics. Kappa range of 0.00–0.20 is quantified as poor strength of agreement, 0.21–0.40 can be seen as fair agreement, 0.41–0.60 is moderate, 0.61–0.80 good and 0.81–1.00 very good strength of agreement for categorical variables [38]. The Cohen’s Kappa measures agreement or reproducibility during the test and retest for reliability [39].

The internal consistency, which measures how closely related the items are for each domain, was measured using Cronbach’s alpha coefficients at Time 1 and Time 2 (1 week). Values of 0.7–0.9 indicate strong internal consistency, while values of 0.5–0.69 are considered acceptable [36, 37]. Corrected item–total correlations with values of > 0.20 indicate that each questionnaire item is correlated with the total score [40]. Values of < 0.2 indicate low correlation and the item may be considered for removal. By referring to the Cronbach-alpha if the item is deleted values, it can be determined if the exclusion of the item would enhance the overall reliability of the instrument [40]. Prior to proceeding with the analysis, missing data were checked. The level of significance was defined as α = 0.05. All statistical tests were two-tailed. All calculations and analyses were carried out using IBM Statistical Package for Social Science version 28 (SPSS Inc., Chicago, IL, USA) program.

## Results

### Sample characteristics for validation study

**Fig. 1:**
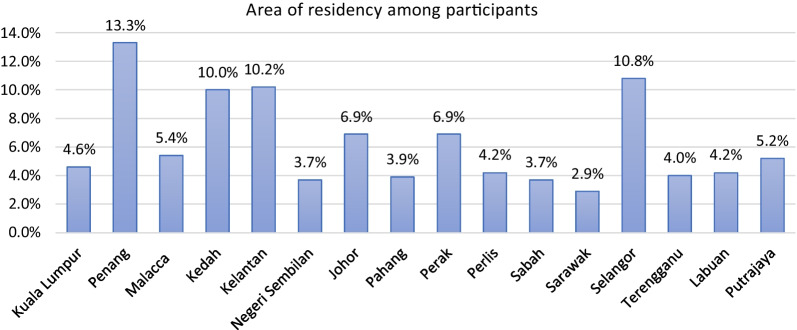
Area of residency among participants (*N* = 519)

A total of 519 participants completed the survey instrument with a response rate of 74.1%. About half of them were females (50.3%), with a median age of 50 years. The majority were married (58.0%), had children (63.6%), were from Penang (13.3%), had a bachelor’s degree (41.2%), and were employed (44.3%). Table 1 summarizes the characteristics data of all participants, and Fig. 1 shows the area of residency for participants.

**Table 1:** Participants demographics (*N* = 519)

Variables	*n* (%)
Age (years), median (IQR)	50.0 (31.0)
Gender	
Female	261 (50.3%)
Male	258 (49.7%)
Marital status	
Single	159 (30.6%)
Married	301 (58.0%)
Divorced	26 (5.0%)
Widowed	33 (6.4%)
Having children	
Yes	330 (63.6%)
No	189 (36.4%)
Education level	
No formal education	23 (4.4%)
Primary school	35 (6.7%)
High school	129 (24.9%)
Diploma	76 (14.6%)
Pre-University	27 (5.2%)
Bachelor’s degree	214 (41.2%)
Master’s or Ph.D. degree	15 (2.9%)
Employment	
Employed	230 (44.3%)
Non-employed	174 (33.5%)
Retired	115 (22.2%)
Health professional	
Yes	40 (7.7%)
No	479 (92.3%)
Student	
Yes	108 (20.8%)
No	411 (79.2%)

*IQR* interquartile range

### Validity of the study instrument

The expert panel found the study instrument’s face and content validity were satisfactory. In addition, all participants of the pilot study completed the questionnaire smoothly without any assistance and did not suggest any changes or comments. As a result, the final questionnaire was used without any further revisions.

### Reliability of the study instrument

Table 2 summarizes the psychometric properties of the Malay version of the drive-thru community pharmacy service questionnaire. All perceptions statements were examined by Cohen’s Kappa. Most of the statements had a very good agreement, as majority of the kappa coefficients were between 0.81 and 1.0 with only one statement (the 2nd statement of believed advantages part) had good agreement kappa coefficient value as it was 0.670, and one statement (the 6th statement of believed disadvantages part) had moderate kappa coefficient value as it was 0.551.

**Table 2:** Psychometric properties of the Malay version of the drive-thru community pharmacy service questionnaire

Domain	Questions	Test (*N* = 32)			Re-test (*N* = 32)
		Cronbach’s Alpha	Cronbach’s alpha if item is deleted	Corrected item—total correlation	Cohen’s Kappa
Perceptions toward drive-thru community pharmacy service as an impact of COVID-19 or at later stage	1. I believe the introduction of drive-thru service makes the community pharmacy services more efficient	0.833	0.828	0.381	1.000
	2. I think that excellent community pharmacies should have driven-thru service during COVID-19 time		0.826	0.419	0.949
	3. I believe that drive-thru community pharmacy service is a friendly service provided by the pharmacy during COVID-19 time or even later on		0.824	0.531	0.882
	4. I believe that drive-thru community pharmacy service may improve my satisfaction with the pharmacy profession		0.822	0.562	0.892
	5. I am supportive to create community pharmacies with drive-thru services all over Malaysia		0.825	0.468	1.000
How do you feel the image of the community pharmacists will be affected by the introduction of drive-thru service?	1. Community pharmacists will appear more concerned with making money than with the health of their patients		0.847	− 0.016^a^	0.915
	2. Community pharmacists will have a good balance between the health of patients and the business side of their work		0.829	0.308	0.888
	3. Community pharmacists will appear more concerned with the health of patients than with the business side of their work		0.845	− 0.121^a^	0.907
Differences between the drive-thru community pharmacy service and in-store drug refill services	1. The prescription might be filled more quickly in drive-thru compared to in-store refill		0.826	0.436	1.000
	2. Pharmacists might be less available to answer questions using drive-thru service compared to in-store refill		0.826	0.418	0.907
	3. Written information might be less supplied using drive-thru pharmacy service compared to in-store refill		0.829	0.348	0.917
	4. Pharmacists cannot explain important points about prescriptions while providing drive-thru service compared to that in-store refill		0.828	0.368	0.956
	5. Drive-thru service provides accessibility and convenience to customers more than the in-store service, especially during COVID-19 time		0.827	0.369	1.000
	6. Unlike in-store service, drive-thru service is suitable only for refill prescriptions but not for new prescriptions		0.829	0.336	1.000
	7. Unlike in-store service, drive-thru service is suitable only for OTC but not for prescriptions medications		0.836	0.165^a^	1.000
Believed advantages toward the drive-thru community pharmacy service as an impact of COVID-19	1. Drive-thru community pharmacy service may help me get my medications on time without delay		0.823	0.501	1.000
	2. Drive-thru community pharmacy will be helpful during COVID-19 time and quarantine time		0.820	0.652	0.670
	3. Drive-thru community pharmacy service has the advantage of serving sick patients, elderly, or disabled people during COVID-19 time		0.825	0.472	0.946
	4. Drive-thru pharmacy service enhances social distancing and reduces the spread of the COVID-19 virus		0.821	0.627	0.946
	5. Drive-thru community pharmacy service reduces the pressure on health care centers during COVID-19 time		0.821	0.573	0.854
	6. Drive-thru community pharmacy service is needed to be implemented in most community pharmacies during COVID-19 time or even later on for getting medications or supplies		0.825	0.444	0.950
Believed disadvantages toward the drive-thru community pharmacy service	1. Drive-thru community pharmacy service may contribute to dispensing errors due to the fast service provided		0.822	0.489	0.867
	2. Drive-thru community pharmacy service may contribute to communication errors between the patient and pharmacist		0.826	0.416	0.904
	3. Drive-thru community pharmacy service may need extra money to offer drive-thru windows		0.834	0.171^a^	0.908
	4. Drive-thru community pharmacy service is not convenient in providing drug information/counselling to patients (especially written information)		0.828	0.358	1.000
	5. Getting prescriptions dispensed as quickly as possible using drive-thru community pharmacy service, the quality of pharmacy service will drop		0.841	0.009^a^	1.000
	6. Drive-thru community pharmacy service restricts the opportunity for interaction with the pharmacist, because the customer feels they cannot ask questions, while they are being hurried through		0.819	0.657	0.551
	7. Drive-thru community pharmacy service restricts the opportunity for interaction with the pharmacist, because the pharmacist will not be able to offer any level of interaction		0.825	0.444	0.949

^a^Corrected item—total correlations less than 0.2

The questionnaire had strong overall consistency, as Cronbach’s alpha was 0.833. Only 5 statements had corrected item–total correlations less than 0.2. However, removing these items did not significantly improve Cronbach’s alpha, hence, they were kept in the analysis. These items were valuable in assessing the perceptions of participants toward the drive-thru community pharmacy service during COVID-19.

### Awareness

The majority of participants (40.1%, *n* = 208) only visited one community pharmacy in the previous month. The participants' most frequent visits to community pharmacies were for the purchase of OTC (over the counter) medicines (55.7%, *n* = 289), followed by the acquisition of COVID-19 prevention supplies, including masks and hygiene items (46.8%, *n* = 243). Only around 10.4% (*n* = 54) of the participants said they had previously used this service, and 9.4% (*n* = 49) said they had a positive experience. Less than half of the participants (46.2%) said they were unaware of the existence of a drive-thru community pharmacy service in their city. Many participants reported that they did not know any information about drive-thru community pharmacy service (35.5%, *n* = 184). The main sources that made participants aware of the drive-thru community pharmacy service were friends or colleagues and the internet (33.5%, *n* = 174), (32.6%, *n* = 169), respectively. Table 3 summarizes the awareness of participants regarding drive-thru community pharmacy service.

**Table 3:** Attitudes toward drive-thru community pharmacy service (*N* = 519)

Variables	*n* (%)
Number of community pharmacies visited last month	
None	120 (23.1%)
One pharmacy	208 (40.1%)
Two pharmacies	126 (24.3%)
Three or more pharmacies	65 (12.5%)
Reasons to visit the community pharmacy^a^	
Over-the-counter medications (OTC)	289 (55.7%)
For beauty products	63 (12.1%)
Prescribed medications	127 (24.5%)
Medical device	120 (23.1%)
Medical consultation	108 (20.8%)
Kid supply	43 (8.3%)
COVID-19 prevention supplies, such as masks and hygiene products	243 (46.8%)
Others	38 (7.3%)
Which category will benefit the most from drive-thru community pharmacy service?	
All population	394 (75.9%)
Women	17 (3.3%)
Geriatrics	61 (11.8%)
People with disabilities	47 (9.1%)
Presence of a drive-thru community pharmacy at your city	
Yes	106 (20.4%)
No	170 (32.8%)
Do not know	243 (46.8%)
If yes, have you tried drive-thru community pharmacy services?	
Yes	54 (10.4%)
No	176 (33.9%)
Not applicable	289 (55.7%)
If yes, how do you evaluate your experience with drive-thru community pharmacy services?	
Excellent	26 (5.0%)
Good	49 (9.4%)
Fair	18 (3.5%)
Poor	0 (0.0%)
Not applicable	426 (82.1%)
If you are going to request an order at a community pharmacy using drive-thru service, what is your preferred method to do that order?	
Through a drive-thru window	171 (32.9%)
Through WhatsApp	153 (29.5%)
Over the phone	86 (16.6%)
Online through application	98 (18.9%)
Through email	11 (2.1%)
If you are going to use a drive-thru service at a community pharmacy, what is your preferred method to get information about your medications(counselling)?^a^	
Briefly through the drive-thru window	262 (50.5%)
Printed brochure given with the order	174 (33.5%)
Written on WhatsApp	240 (46.2%)
Verbally over the phone	164 (31.6%)
Through a personal visit	193 (37.2%)
Through email	47 (9.1%)
Where did you get information regarding the drive-thru community pharmacy^a^	
Pharmacy staff	129 (24.9%)
Doctors	67 (12.9%)
Leaflets	67 (12.9%)
Television	74 (14.3%)
Internet	169 (32.6%)
Friends or colleagues	174 (33.5%)
Do not know	184 (35.5%)
Are you supportive to establish drive-thru services at community pharmacies?	
Yes	511 (98.5%)
No	8 (1.5%)

^a^Respondents could pick more than one answer (percentage summation ≠ 100%)

### Attitudes

Most of the participants were in favor of establishing drive-thru service at community pharmacies in the country 98.5% (*n* = 511) and believed that drive-thru community pharmacy would benefit all population 75.9% (*n* = 394). Participants preferred to use the drive-thru window to place an order at a community pharmacy using the drive-thru service (*n* = 171), and they preferred to receive counseling while using this service through the drive-thru window as well 50.5% (*n* = 262). Table 3 summarizes the attitudes of participants regarding drive-thru community pharmacy service.

### Perceptions

A positive perception was found toward the drive-thru community pharmacy service among the participants (Table 4). Less than half of the participants 41.8% (*n* = 217) agreed that the drive-thru community pharmacy service is a friendly service provided by the pharmacy during COVID-19 time or even at later stage; and that the drive-thru community pharmacy service may improve participants’ satisfaction with the pharmacy profession. Most of the participants 47.2% (*n* = 245) agreed that community pharmacists will have a good balance between the health of patients and the business side of their work.

**Table 4:** Perceptions toward drive-thru community pharmacy service (N = 519)

Variables	Strongly disagree n (%)	Disagree *n* (%)	Neutral *n* (%)	Agree *n* (%)	Strongly agree *n* (%)
Perceptions toward drive-thru community pharmacy services an impact of COVID-19 or even at later stage					
I believe the introduction of drive-thru service makes the community pharmacy services more efficient	1 (0.2%)	2 (0.4%)	48 (9.2%)	245 (47.2%	223 (43.0%)
I believe that drive-thru community pharmacy service is a friendly service provided by the pharmacy during COVID-19 time or even at later stage	1 (0.2%)	5 (1.0%)	41 (7.9%)	217 (41.8%)	255 (49.1%)
I believe that drive-thru community pharmacy service may improve my satisfaction with the pharmacy profession	2 (0.4%)	1 (0.2%)	58 (11.2%)	217 (41.8%)	241 (46.4%)
I am supportive of the introduction of drive-thru service to community pharmacy practice during COVID-19 time	2 (0.4%)	7 (1.3%)	63 (12.1%)	230 (44.3%)	217 (41.8%)
I am supportive to create community pharmacies with drive-thru services all over Malaysia	1 (0.2%)	4 (0.8%)	48 (9.2%)	207 (39.9%)	259 (49.9%)
How do you feel the image of the community pharmacists will be affected by the introduction of drive-thru services?					
Community pharmacists will appear more concerned with making money than with the health of their patients^a^	52 (10.0%)	151 (29.1%)	146 (28.1%)	116 (22.4%)	54 (10.4%)
Community pharmacists will have a good balance between the health of patients and the business side of their work	6 (1.2%)	31 (6.0%)	124 (23.9%)	245 (47.2%)	113 (21.8%)
Community pharmacists will appear more concerned with the health of patients than with the business side of their work	15 (2.9%)	46 (8.9%)	162 (31.2%)	198 (38.2%)	98 (18.9%)
Differences between the drive-thru community pharmacy service and in-store drug refill services					
The prescription might be filled more quickly in drive-thru compared to in-store refill	2 (0.4%)	11 (2.1%)	70 (13.5%)	267 (51.4)	169 (32.6%)
Pharmacists might be less available to answer questions using drive-thru service compared to in-store refill^a^	14 (2.7%)	57 (11.0%)	132 (25.4%)	216 (41.6%)	100 (19.3%)
Written information might be less supplied using drive-thru pharmacy service compared to in-store refill^a^	10 (1.9%)	61 (11.8%)	111 (21.4%)	230 (44.3%)	107 (20.6%)
Pharmacists cannot explain important points about prescriptions while providing drive-thru service compared to that in-store refill^a^	13 (2.5%)	60 (11.6%)	112 (21.6%)	231 (44.5%)	103 (19.8%)
Drive-thru service provides accessibility and convenience to customers more than the in-store service, especially during COVID-19 time	4 (0.8%)	11 (2.1%)	85 (16.4%)	252 (48.6%)	167 (32.2%)
Unlike in-store service, drive-thru service is suitable only for refill prescriptions but not for new prescriptions^a^	7 (1.3%)	33 (6.4%)	105 (20.2%)	205 (39.5%)	169 (32.6%)
Unlike in-store service, drive-thru service is suitable only for OTC but not for prescriptions medications^a^	11 (2.1%)	47 (9.1%)	122 (23.5%)	198 (38.2%)	141 (27.2%)

^a^Reversed coded statements

### Differences between drive-thru and in-store drug refills

About 51.4% of the participants agreed that the prescription might be filled more quickly in drive-thru compared to in-store pharmacy services, and 48.6% agreed that drive-thru service is more accessible and convenient compared to in-store pharmacy services, especially during COVID-19 time. However, participants agreed with the following statements about the drive-thru service: the pharmacist is less accessible to customers' questions (41.6%, *n* = 216), offers less written information (44.3%, *n* = 230), and is unable to clarify important points of prescriptions (44.5%, *n* = 231). Many participants thought that the drive-thru service is only suitable for refill prescriptions (39.5%, *n* = 205), and for getting OTC products (38.2%, *n* = 198). All details for participants-perceived differences between drive-thru and in-store drug refills are summarized in Table 4.

### Believed advantages and disadvantages toward drive-thru community pharmacy service during COVID-19

The two most highly agreed-upon advantages of the drive-thru community pharmacy service were improving social distance and minimizing COVID-19 virus spread by 47.0% (*n* = 244), followed by providing a valuable service for ill patients, the elderly, or individuals with disabilities at the time (45.3%, *n* = 235). The need for additional funding to provide drive-thru windows (21.2%, *n* = 110), followed by limiting the opportunity for interaction with the pharmacist, because the customer feels that they cannot ask questions, while they are being hurried through (20.2%, *n* = 105), were the two strongly agreed disadvantages of drive-thru community pharmacy service. Table 5 lists all further identified advantages and disadvantages of drive-thru community pharmacy service.

**Table 5:** Believed advantages and disadvantages toward drive-thru community pharmacy service (*N* = 519)

	Strongly disagree *n* (%)	Disagree *n* (%)	Neutral *n* (%)	Agree *n* (%)	Strongly agree *n* (%)
Believed advantages toward the drive-thru community pharmacy service an impact of COVID-19					
Drive-thru community pharmacy service may help me get my medications on time without delay	4 (0.8%)	5 (1.0%)	53 (10.2%)	256 (49.3%)	201 (38.7%)
Drive-thru community pharmacy will be helpful during COVID-19 time and quarantine time	1 (0.2)	3 (0.6%)	48 (9.2%)	241 (46.4%)	226 (43.5%)
Drive-thru community pharmacy service has the advantage of serving sick patients, elderly, or disabled people during COVID-19 time	4 (0.8)	6 (1.2%)	52 (10.0%)	222 (42.8%)	235 (45.3%)
Drive-thru pharmacy service enhances social distancing and reduces the spread of the COVID-19 virus	3 (0.6)	8 (1.5%)	49 (9.4%)	215 (41.4%)	244 (47.0%)
Drive-thru community pharmacy service reduces the pressure on health care centers during COVID-19 time	4 (0.8%)	12 (2.3%)	71 (13.7%)	234 (45.1%)	198 (38.2%)
Drive-thru community pharmacy service is needed to be implemented in most community pharmacies during COVID-19 time or even at later stage for getting medications or supplies	3 (0.6%)	6 (1.2%)	57 (11.0%)	233 (44.9%)	220 (42.4%)
Believed disadvantages toward the drive-thru community pharmacy services					
Drive-thru community pharmacy service may contribute to dispensing errors due to the fast service provided^a^	17 (3.3%)	70 (13.5%)	138 (26.6%)	221 (42.6%)	73 (14.1%)
Drive-thru community pharmacy service may contribute to communication errors between the patient and pharmacist^a^	10 (1.9%)	65 (12.5%)	149 (28.7%)	214 (41.2%)	81 (15.6%)
Drive-thru community pharmacy service may need extra money to offer drive-thru windows^a^	13 (2.5%)	43 (8.3%)	122 (23.5%)	231 (44.5%)	110 (21.2%)
Drive-thru community pharmacy service is not convenient in providing drug information/counselling to patients (especially written information)^a^	9 (1.7%)	50 (9.6%)	125 (24.1%)	235 (45.3%)	100 (19.3%)
Getting prescriptions dispensed as quickly as possible using drive-thru community pharmacy service, the quality of pharmacy service will drop^a^	22 (4.2%)	96 (18.5%)	133 (25.6%)	185 (35.6%)	83 (16.0%)
Drive-thru community pharmacy service restricts the opportunity for interaction with the pharmacist, because the customer feels they cannot ask questions, while they are being hurried through^a^	13 (2.5%)	59 (11.4%)	112 (21.6%)	230 (44.3%)	105 (20.2%)
Drive-thru community pharmacy service restricts the opportunity for interaction with the pharmacist, because the pharmacist will not be able to offer any level of interaction^a^	12 (2.3%)	66 (12.7%)	113 (21.8%)	225 (43.4%)	103 (19.8%)
Total perceptions score Median (IQR)	92.0 (15.0)				

^a^Reversed coded statements

## Discussion

This study aimed to validate a Malay version of the drive-thru community pharmacy service questionnaire and to obtain a better understanding of the Malaysian public’s awareness, attitudes, and perceptions of drive-thru community pharmacy service during COVID-19. The content and face validity for the drive-thru community pharmacy service Malay version questionnaire were deemed to be satisfactory by the expert panel. In addition, the Malay version questionnaire was accepted during the pilot test among participants in terms of being free of difficulty or ambiguity with no suggested changes. The drive-thru community pharmacy service questionnaire was found to be reliable as most of the statements had very good agreement coefficient values, with strong internal consistency for the whole tool with a strong Cronbach’s alpha of 0.833 [36].

Participants in the current study had a limited level of awareness about the existence of drive-thru pharmacies in a community context, which accounts for the low rate at which they used this service. This is due to the fact that the service was only recently launched in Malaysia in February 2022 [23]. Same findings were shown in previous studies, and most customers in Jordan and Saudi Arabia had little awareness of drive-thru pharmacy services in a community setting [21, 22].

Friends or colleagues, the internet, and pharmacy personnel were the most common sources that made participants aware of the availability of drive-thru community pharmacy service. Jordanian pharmacy consumers using the drive-thru service revealed comparable outcomes, as the main sources that made them aware about this service were friends, the internet, and pharmacy staff [21]. Surprisingly, only 12.9% of the participants in the current survey cited doctors as the source who made them aware of the existence of community pharmacies with drive-thru services. It points out the necessity for doctors to increase public awareness of this service.

Most study participants had favorable attitudes toward the need for drive-thru community pharmacy services; as 49.1% of them were strongly in favor of establishing such services during COVID-19 time or at later stage, 49.9% were strongly in favor of establishing such services throughout Malaysia, and 75.9% thought that such services would be beneficial to the whole population. These results were consistent with a prior study conducted in Saudi Arabia, where the majority of respondents who used the drive-thru community pharmacy service believed that this service would reduce the likelihood of the COVID-19 pandemic and benefit everyone [22]. Furthermore, about 45% of the present participants strongly believed this service will benefit patients who are ill, the elderly, or individuals with disabilities during COVID-19. The same findings, indicating drive-thru pharmacy services are beneficial for a particular population, including geriatrics, the disabled, and sick, were published prior to the emergence of COVID-19 [12, 21].

The drive-thru window was the preferred method among the current participants for placing an order at a community pharmacy using drive-thru services, and for receiving short counselling when using this service. This could be explained by the opinion previously expressed in Taiwan that the drive-thru area had less disturbance and noise while being used [12].

About 21.8% of the participants strongly agreed that community pharmacists will be able to manage their work well between the business side of things and the health of their patients. Similar to this, 20.9% of Jordanians who used a drive-thru pharmacy service thought that local pharmacists would strike a good balance between patient health and the business side of their jobs [21]. This is justified by the dramatic change in pharmacy practice focusing mainly on patients’ care [41, 42].

The perceived differences between drive-thru and in-store drug refills among the study participants were mainly that the prescription might be filled more quickly in drive-thru compared to in-store pharmacy services. Same results were documented previously [12, 21, 22]. Prior to the drive-thru service being implemented in Taiwan, it took at least 40 min to pick up in-store refill prescriptions; compared with only 3 min using drive-thru refill [12]. Community pharmacies’ consumers in Jordan and Saudi Arabia believed that prescriptions might be filled more quickly using drive-thru compared to traditional pharmacy services [21, 22]. On the other hand, participants in this study believed that, in contrast to in-store pharmacy services, those pharmacists providing drive-thru services were less readily available to answer their queries, provided less written information, and were unable to clarify crucial points of prescriptions. These results are supported by earlier research, which showed that interactions between patients and pharmacists were better when prescriptions were filled in-store [43], and that drive-thru service might shorten interactions between customers and pharmacists [21, 44].

According to the current study, participants had favorable perceptions of this service during COVID-19, because they thought it had several advantages. The two most highly agreed advantages of this service during COVID-19 were to encourage social isolation and stop the virus from spreading. Only one study discussed this service during COVID-19 in Saudi Arabia [22]. All previously conducted studies were in line with our findings that drive-thru service is an accessible and convenient service that that facilitates receiving prescriptions promptly and with minimal waiting time [10, 20–22].

This study supported what was documented in the literature about the disadvantages toward this service while using it [20, 21, 43–45]. The participants believed that it may mainly contribute to restricting the opportunity for interaction with the pharmacist, and difficulty in providing drug information/counselling to customers (especially written information) [20, 21, 43–45]. In addition, it was believed among the study participants that drive-thru community pharmacy service is suitable for refilling prescriptions and for getting OTC products. This result is consistent with a previous study in which Jordanian consumers thought that drive-thru pharmacies were only appropriate for OTC purchases and prescription refills [21].

### Strengths and limitations

This research provides the validation and reliability tests to the Malay version of the drive-thru community pharmacy service questionnaire including the accessibility, pharmacist–patient interaction, and the perceived advantages and concerns depicted by the Malaysian public in the Malay language; thus, to better inform future pandemic preparation efforts, including drive-thru pharmacies in Malaysia. It is crucial to acknowledge certain limitations that may influence the direct translation of our findings into broader policy and practice. First, to avoid the spread of COVID-19, the instrument was distributed online, and participants completed it using Google Forms; as a result, replies from regions without access to the internet may not have been recorded. Second, the scope of our research, focused on Malaysian population only and it may has limited the generalizability of the results to diverse settings. Besides, the reliance on self-reported data in this study may introduce a response bias. Third, the test–retest reliability had a small sample size. As a result, the dependability test results should be interpreted with caution. This was mostly, because it was challenging to obtain second sets of responses, because they were provided voluntarily and without financial benefits; therefore, we relied only on participants’ cooperation. Fourth, there was no other validated instrument for comparison to do criterion and construct validity. Finally, the drive-thru community pharmacy service has not been the subject of many previous studies, and those that have been conducted have solely focused on Malaysian governmental hospitals, such as Queen Elizabeth Hospital and Hospital Raja Perempuan Zainab II [19, 20]. Nevertheless, this research provides an insight to the drive-thru pharmacies in Malaysia, thus, it is important to interpret the findings within the context of these acknowledged limitations.

Further studies are needed to assess the test–retest reliability of the translated drive-thru community pharmacy service questionnaire on a larger sample size. In addition, future studies discussing the economic side by analyzing the full cost of implementing drive-thru community pharmacy service are recommended.

## Conclusion

This study showed that the translated Malay version of drive-thru community pharmacy service during the COVID-19 questionnaire is valid and reliable to assess the perceptions toward drive-thru community pharmacy service during COVID-19 in Malaysia. The questionnaire had strong internal consistency. Most of the statements showed very good agreement. In addition, Malaysian public expressed good awareness, and favorable attitudes and perceptions toward drive-thru community pharmacy service during COVID-19.

### Supplementary Information

**Additional file 1:** The English version of the questionnaire.

**Additional file 2:** The Malay version of the questionnaire.

## Data Availability

The original contributions presented in the study are included in the article/supplementary material, further inquiries can be directed to the corresponding author/s. All data generated or analyzed during this study are included in this article [and its Additional files].
