# Supplementary material for: Reliability and validity of the Malay version of the drive-thru community pharmacy service questionnaire and the Malaysian public’s awareness, attitudes, and perceptions of drive-thru community pharmacy during COVID-19
Source: J Pharm Policy Pract. 2023 Nov 28;16:159. doi: 10.1186/s40545-023-00666-6 (PMC10683268; doi:10.1186/s40545-023-00666-6)
Supplement: Supplementary file 1 — Additional file 1: The English version of the questionnaire. [file 40545_2023_666_MOESM1_ESM.pdf]

## Appendix 1. The English version of the questionnaire.

**First part: Demographics.**

Please for this part choose only one option.

| | | | | | | |
|---|---|---|---|---|---|---|
| **Age (Years) write your age** | | | | | | |
| **Gender** | Male | Female | | | | |
| **Marital Status** | Single | Married | Divorced | Widowed | | |
| **Having children** | Yes | No | | | | |
| **Area of residency** | Kuala Lumpur | Penang | Malacca | Kedah | Kelantan | Negeri Sembilan |
| | Johor | Pahang | Perak | Perlis | Sabah | Sarawak |
| | Selangor | Terengganu | Labuan | Putrajaya | | |
| **Educational level** | No formal education | Primary School | High School | Diploma | Pre-University | Bachelor's degree |
| | Master's or Ph.D. degree | | | | | |
| **Employment status** | Employed | Non-employed | Retired | | | |
| **Are you from the medical team (e.g., doctors, nurses, pharmacists, allied health professionals)?** | Yes | No | | | | |
| **Are you a full-time or part-time student?** | Yes | No | | | | |

**Second part: Attitudes towards community pharmacy drive-thru service.**

***<u>Please be aware that we are asking about community pharmacies, not hospital pharmacies.</u>***

| **Attitudes towards drive-thru community pharmacy service** |
|---|
| **The number of community pharmacies visited last month** |
| 1. None |
| 2. One pharmacy |
| 3. Two pharmacies |
| 4. Three or more pharmacies |
| **Reasons to visit the community pharmacy (more than 1 option could be chosen)** |
| 1. Over the counter medications |
| 2. For beauty products |
| 3. Prescribed medications |
| 4. Medical Device |
| 5. Medical consultation |
| 6. Kid supply |
| 7. COVID-19 prevention supplies such as masks and hygiene products. |
| 8. Others (……………………………) |
| **Which category will benefit the most from drive-thru community pharmacy service?** |
| 1. All population |
| 2. Women |
| 3. Geriatrics |
| 4. People with disabilities |
| **Presence of drive-thru community pharmacy at your city** |
| 1. Yes |
| 2. No |
| 3. Don't know |
| **If yes, have you tried drive-thru community pharmacy service?** |
| 1. Yes |
| 2. No |
| 3. Not applicable |
| **If yes, how do you evaluate your experience with drive-thru community pharmacy service?** |
| 1. Excellent |
| 2. Good |
| 3. Fair |
| 4. Poor |
| 5. Not applicable |
| **If you are going to request an order at a community pharmacy using drive-thru service, what is your preferred method to do that order?** |
| 1- Through a drive-thru window |
| 2- Through WhatsApp |
| 3- Over the phone |
| 4- Online through application |
| 5- Through email |

| |
|---|
| **If you are going to use a drive-thru service at a community pharmacy, what is your preferred method to get information about your medications(counselling)? (more than 1 option could be chosen)** |
| 1- Briefly through the drive-thru window |
| 2- Printed brochure given with the order |
| 3- Written on WhatsApp |
| 4- Verbally over the phone |
| 5- Through a personal visit |
| 6- Through email |
| **Where did you get information regarding drive-thru community pharmacy (more than 1 option could be chosen)** |
| 1. Pharmacy staff |
| 2. Doctors |
| 3. Leaflets |
| 4. Television |
| 5. Internet |
| 6. Friends or Colleagues |
| 7. Don't know |
| **Are you supportive to establish drive-thru service at community pharmacies?** |
| 1. Yes |
| 2. No |

**Third part: Perceptions towards community pharmacy drive-thru service.**

Please read the following statements, and **indicate your degree of agreement or disagreement for each statement based on the following Likert scale(strongly disagree, disagree, neutral, agree, strongly agree).**

| **Perceptions towards drive-thru community pharmacy service as an impact of COVID-19 or at later stage** | **Strongly agree** | **Agree** | **Neutral** | **Disagree** | **Strongly disagree** |
|---|---|---|---|---|---|
| 1. I believe the introduction of drive-thru service makes the community pharmacy services more efficient. | | | | | |
| 2. I believe that drive-thru community pharmacy service is a friendly service provided by the pharmacy during COVID-19 time at later stage. | | | | | |
| 3. I believe that drive-thru community pharmacy service may improve my satisfaction with the pharmacy profession. | | | | | |
| 4. I am supportive of the introduction of drive-thru service to community pharmacy practice during COVID-19 time. | | | | | |
| 5. I am supportive to create community pharmacies with drive-thru services all over Malaysia. | | | | | |
| **How do you feel the image of the community pharmacists will be affected by the introduction of drive-thru service?** | | | | | |
| 1. Community pharmacists will appear more concerned with making money than with the health of their patients. | | | | | |
| 2. Community pharmacists will have a good balance between the health of patients and the business side of their work. | | | | | |

| | | | | | |
|---|---|---|---|---|---|
| 3. Community pharmacists will appear more concerned with the health of patients than with the business side of their work. | | | | | |
| **Differences between the drive-thru community pharmacy service and in-store drug refill services.** | | | | | |
| 1. The prescription might be filled more quickly in drive-thru compared to in-store refill. | | | | | |
| 2. Pharmacists might be less available to answer questions using drive-thru service compared to in-store refill. | | | | | |
| 3. Written information might be less supplied using drive-thru pharmacy service compared to in-store refill. | | | | | |
| 4. Pharmacists cannot explain important points about prescriptions while providing drive-thru service compared to that in-store refill. | | | | | |
| 5. Drive-thru service provides accessibility and convenience to customers more than the in-store service, especially during COVID-19 time. | | | | | |
| 6. Unlike in-store service, drive-thru service is suitable only for refill prescriptions but not for new prescriptions. | | | | | |
| 7. Unlike in-store service, drive-thru service is suitable only for OTC but not for prescriptions medications. | | | | | |
| **Believed advantages towards the drive-thru community pharmacy service as an impact of COVID-19** | | | | | |
| 1. Drive-thru community pharmacy service may help me get my medications on time without delay. | | | | | |
| 2. Drive-thru community pharmacy will be helpful during COVID-19 time and quarantine time. | | | | | |
| 3. Drive-thru community pharmacy service has the advantage of serving sick patients, elderly, or disabled people during COVID-19 time. | | | | | |
| 4. Drive-thru pharmacy service enhances social distancing and reduces the spread of the COVID-19 virus. | | | | | |
| 5. Drive-thru community pharmacy service reduces the pressure on healthcare centers during COVID-19 time. | | | | | |
| 6. Drive-thru community pharmacy service is needed to be implemented in most community pharmacies during COVID-19 time or at later stage for getting medications or supplies. | | | | | |
| **Believed disadvantages towards the drive-thru community pharmacy service** | | | | | |
| 1. Drive-thru community pharmacy service may contribute to dispensing errors due to the fast service provided. | | | | | |
| 2. Drive-thru community pharmacy service may contribute to communication errors between the patient and pharmacist. | | | | | |
| 3. Drive-thru community pharmacy service may need extra money to offer drive-thru windows. | | | | | |

| | | | | | |
|---|---|---|---|---|---|
| 4. Drive-thru community pharmacy service is not convenient in providing drug information/counselling to patients (especially written information). | | | | | |
| 5. Getting prescriptions dispensed as quickly as possible using drive-thru community pharmacy service, the quality of pharmacy service will drop. | | | | | |
| 6. Drive-thru community pharmacy service restricts the opportunity for interaction with the pharmacist because the customer feels they can't ask questions while they're being hurried through. | | | | | |
| 7. Drive-thru community pharmacy service restricts the opportunity for interaction with the pharmacist because the pharmacist will not be able to offer any level of interaction. | | | | | |
