# Supplementary material for: Reliability and validity of the Malay version of the drive-thru community pharmacy service questionnaire and the Malaysian public’s awareness, attitudes, and perceptions of drive-thru community pharmacy during COVID-19
Source: J Pharm Policy Pract. 2023 Nov 28;16:159. doi: 10.1186/s40545-023-00666-6 (PMC10683268; doi:10.1186/s40545-023-00666-6)
Supplement: Supplementary file 2 — Additional file 2: The Malay version of the questionnaire. [file 40545_2023_666_MOESM2_ESM.pdf]

Appendix 2. The Malay version of the questionnaire.

Bahagian Pertama: Demografik.

Sila pilih satu jawapan bagi soalan-soalan di bawah

**Umur (Tahun)**						
**Jantina**	Lelaki	Perempuan				
**Status perkahwinan**	Bujang	Berkahwin	Bercerai	Janda/Duda		
**Mempunyai anak**	Ya	Tidak				
**Negeri kediaman**	Kuala Lumpur	Pulau Pinang	Melaka	Kedah	Kelantan	Negeri Sembilan
	Johor	Pahang	Perak	Perlis	Sabah	Sarawak
	Selangor	Terengganu	Labuan	Putrajaya		
**Tahap pendidikan**	Tiada pendidikan formal	Sekolah rendah	Sekolah menengah	Diploma	Pra-Universiti	Sarjana Muda
	Sarjana lanjutan atau Doktor falsafah					
**Status pekerjaan**	Bekerja	Tidak bekerja	Bersara			
**Adakah anda bekerja di sektor kesihatan (contohnya, doktor, jururawat, pegawai farmasi, pegawai kesihatan bersekutu)?**	Ya	Tidak				
**Adakah anda pelajar sepenuh masa atau separuh masa?**	Ya	Tidak				

Bahagian kedua: Sikap terhadap perkhidmatan farmasi komuniti pandu-lalu.

Makluman. Soalan-soalan di bawah adalah berkenaan farmasi di komuniti, dan bukan farmasi di hospital

**Sikap terhadap perkhidmatan farmasi komuniti pandu-lalu**
**Bilangan farmasi komuniti yang dikunjungi dalam masa sebulan yang lalu**
1. Tiada
2. Satu
3. Dua
4. Tiga atau lebih
**Tujuan mengunjungi farmasi komuniti (Dibenarkan memilih lebih daripada 1 jawapan)**
1. Mendapatkan ubat belian kaunter (*over the counter medication*)
2. Mendapatkan produk kecantikan
3. Mendapatkan preskripsi ubat
4. Mendapatkan peralatan perubatan
5. Mendapatkan nasihat perubatan
6. Mendapatkan keperluan bayi dan kanak-kanak
7. Mendapatkan keperluan pencegahan COVID-19 seperti pelitup muka & produk pembasmi kuman
8. Lain-lain
**Kategori manakah yang paling mendapat manfaat daripada pandu-lalu di farmasi komuniti?**
1. Semua golongan
2. Wanita
3. Warga emas
4. Orang kelainan upaya (OKU)
**Kewujudan perkhidmatan farmasi komuniti pandu-lalu di bandar anda**
1. Ya
2. Tidak
3. Tidak tahu
**Jika Ya, pernahkah anda menggunakan perkhidmatan pandu-lalu di farmasi komuniti?**
1. Ya
2. Tidak
3. Tidak berkaitan
**Jika Ya, bagaimana anda menilai pengalaman anda berurusan dengan farmasi komuniti pandu-lalu?**
1. Sangat baik
2. Baik
3. Memuaskan
4. Tidak memuaskan
5. Tidak berkaitan
**Jika anda ingin membuat pesanan di farmasi komuniti, dengan menggunakan perkhidmatan pandu-lalu, apakah kaedah yang paling anda sukai?**
1. Melalui tingkap pandu-lalu
2. Melalui aplikasi *Whatsapp*
3. Melalui telefon
4. Di atas talian melalui aplikasi telefon
5. Melalui emel
**Jika anda menggunakan khidmat pandu-lalu di farmasi komuniti, apakah kaedah yang anda gemari untuk mendapatkan maklumat tentang ubat-ubatan anda (kaunseling)? (Dibenarkan memilih lebih daripada 1 jawapan)**

1. Penerangan ringkas melalui tingkap pandu-lalu
2. Cetakan risalah ringkas yang diberi bersama pesanan
3. Penerangan melalui aplikasi *Whatsapp*
4. Penerangan melalui panggilan telefon
5. Penerangan terus secara langsung
6. Penerangan melalui emel
**Dari mana anda mendapat maklumat berkenaan perkhidmatan pandu-lalu di farmasi komuniti (Dibenarkan memilih lebih daripada 1 jawapan)**
1. Pegawai farmasi
2. Doktor
3. Cetakan risalah ringkas
4. Televisyen
5. Internet
6. Kawan-kawan atau rakan sekerja
7. Tidak tahu
**Adakah anda menyokong pelaksanaan perkhidmatan pandu-lalu di farmasi komuniti?**
1. Ya
2. Tidak

Bahagian ketiga: Persepsi terhadap perkhidmatan pandu-lalu di farmasi komuniti.

Sila fahami kenyataan-kenyataan di bawah, dan **tandakan jawapan bagi menunjukkan tahap persetujuan anda untuk setiap kenyataan tersebut, menggunakan Likert scale seperti di bawah (sangat tidak bersetuju, tidak bersetuju, neutral, bersetuju, sangat bersetuju).**

**Persepsi terhadap perkhidmatan pandu-lalu di farmasi komuniti akibat kesan dari COVID-19 atau pada masa hadapan**	**Sangat Bersetuju**	**Bersetuju**	**Neutral**	**Tidak Bersetuju**	**Sangat Tidak Bersetuju**
1. Saya percaya bahawa pengenalan perkhidmatan pandu-lalu akan meningkatkan tahap kecekapan di farmasi komuniti.					
2. Saya merasakan bahawa farmasi komuniti yang bagus perlu mempunyai perkhidmatan pandu-lalu tempoh COVID-19.					
3. Saya percaya bahawa perkhidmatan pandu-lalu di farmasi komuniti adalah suatu perkhidmatan yang mesra pelanggan sepanjang tempoh COVID-19 atau pada masa hadapan.					
4. Saya percaya bahawa perkhidmatan pandu-lalu di farmasi komuniti dapat meningkatkan tahap kepuasan saya terhadap perkhidmatan farmasi.					
5. Saya menyokong pembukaan farmasi komuniti yang menyediakan perkhidmatan pandu-lalu di seluruh Malaysia					
**Pada pandangan anda, bagaimanakah imej pegawai farmasi di farmasi komuniti akan terkesan disebabkan pengenalan perkhidmatan pandu-lalu?**					
1. Pegawai farmasi akan kelihatan lebih mengutamakan keuntungan jualan berbanding dengan tahap kesihatan pesakit mereka.					

2. Pegawai farmasi akan dapat mengimbangkan daripada kedua-dua sudut, iaitu daripada tahap kesihatan pesakit mereka dan juga daripada sudut pengurusan perniagaan mereka.					
3. Pegawai farmasi akan kelihatan lebih mengutamakan tahap kesihatan pesakit mereka berbanding dengan keuntungan jualan.					
**Perbezaan diantara perkhidmatan pandu-lalu di farmasi komuniti berbanding dengan perkhidmatan bekalan ubat di dalam bangunan fasiliti**					
1. Preskripsi dapat dibekalkan dengan lebih cepat melalui pandu-lalu berbanding dengan di dalam bangunan fasiliti.					
2. Pegawai farmasi kurang menjawab soalan daripada pelanggan di pandu-lalu berbanding di dalam bangunan fasiliti.					
3. Maklumat tambahan secara bercetak/bertulis mungkin kurang diberikan di pandu-lalu berbanding di dalam bangunan fasiliti.					
4. Pegawai farmasi tidak dapat menerangkan maklumat penting tentang ubat preskripsi semasa di pandu-lalu berbanding di dalam bangunan fasiliti.					
5. Perkhidmatan pandu-lalu memberi kemudahan dan khidmat farmasi kepada pelanggan berbanding di dalam bangunan fasiliti, terutamanya sepanjang tempoh COVID-19.					
6. Perkhidmatan pandu-lalu sesuai hanya untuk pengambilan ubat preskripsi ulangan, tetapi tidak sesuai untuk pengambilan ubat preskripsi yang baru.					
7. Perkhidmatan pandu-lalu sesuai hanya untuk ubat belian kaunter (*over the counter*), tetapi tidak sesuai untuk ubat preskripsi.					
**Kelebihan farmasi komuniti pandu-lalu kerana kesan COVID-19**					
1. Perkhidmatan pandu-lalu dapat membantu saya mendapatkan bekalan ubat secara berterusan tanpa kelewatan.					
2. Farmasi komuniti yang mempunyai khidmat pandu-lalu sangat membantu penduduk semasa tempoh COVID-19 dan tempoh kuarantin.					
3. Perkhidmatan pandu-lalu mempunyai kelebihan untuk membantu golongan yang sakit, warga emas, atau warga OKU semasa tempoh COVID-19.					
4. Perkhidmatan pandu-lalu membantu dalam penjarakan sosial dan mampu mengurangkan penyebaran virus COVID-19.					
5. Perkhidmatan pandu-lalu dapat mengurangkan beban tugas yang ditanggung oleh fasiliti kesihatan seperti klinik kesihatan semasa tempoh COVID-19.					
6. Perkhidmatan pandu-lalu amat diperlukan hampir di semua farmasi komuniti semasa tempoh COVID-19					

atau di masa hadapan untuk mendapatkan ubat-ubatan atau bekalan.					
**Kelemahan farmasi komuniti pandu-lalu**					
1. Perkhidmatan pandu-lalu menyumbang kepada kesilapan semasa pendispensan ubat disebabkan oleh perkhidmatan yang pantas.					
2. Perkhidmatan pandu-lalu menyumbang kepada kesalahan dari segi komunikasi di antara pesakit dan pegawai farmasi.					
3. Perkhidmatan pandu-lalu memerlukan peruntukan tambahan bagi menyediakan tingkap untuk pandu-lalu.					
4. Perkhidmatan pandu-lalu menyebabkan kesukaran dalam memberikan maklumat tentang ubat atau dalam menjalankan sesi kaunseling kepada pesakit (terutamanya maklumat dalam bentuk bertulis).					
5. Ubat preskripsi yang dibekalkan kepada pesakit secara pantas menggunakan perkhidmatan pandu-lalu, boleh menurunkan kualiti perkhidmatan farmasi.					
6. Perkhidmatan pandu-lalu akan mengehadkan peluang untuk berinteraksi bersama pegawai farmasi kerana pelanggan merasakan mereka tidak dapat menujukan soalan, ketika mana urusan mereka dilakukan secara tergesa-gesa.					
7. Perkhidmatan pandu-lalu akan mengehadkan peluang untuk berinteraksi bersama pegawai farmasi kerana pegawai farmasi tidak menyediakan sebarang ruang untuk berinteraksi.					
